# A Case of Severely Impaired Consciousness and Convulsions From Attempted Suicide by Ingesting Triflumizole Emulsion: Clinical Features and Literature Review

**DOI:** 10.7759/cureus.55356

**Published:** 2024-03-01

**Authors:** Kentaro Hayashi, Koji Wake

**Affiliations:** 1 Emergency Medicine, Dokkyo Medical University, Tochigi, JPN; 2 Medicine, Dokkyo Medical University, Tochigi, JPN

**Keywords:** triflumizole, intoxication, impaired consciousness, convulsion, agricultural fungicide

## Abstract

Triflumizole (TFZ) is a fungicide widely used in agriculture to prevent fungal infections of fruits and vegetables. Although it is considered safe for humans and animals, its toxicity profile in humans remains largely unexplored. Here, we describe a case where an individual experienced symptoms suggestive of intoxication after ingesting TFZ emulsion.

A 70-year-old man ingested TFZ emulsion (Trifumin emulsion^TM^) and alcohol in an attempt to commit suicide. He developed a severe disturbance of consciousness, which was not explained by the estimated blood alcohol concentration, and experienced convulsions. We managed this patient with symptomatic treatment, temporary mechanical ventilation, and antiepileptic drugs. He subsequently recovered without any sequelae.

We present the first case of acute oral intoxication with TFZ emulsion. Moreover, we review the literature on TFZ-induced organ dysfunction and discuss the possible mechanisms and management of this condition.

## Introduction

Triflumizole (TFZ, 4-chloro-N-[1-(1H-imidazol-1-yl)-2-propoxyethylidene]-2-(trifluoromethyl)-aniline, C15H15ClF3N3O) [1] is a systemic fungicide that inhibits ergosterol biosynthesis in fungi. It is widely used in agriculture to prevent fungal infections in fruits and vegetables, such as apples, pears, grapes, cucumbers, tomatoes, and peppers. In Japan, it is marketed as Trifumin emulsion™ [2] (Nihon Soda Co., Ltd., Tokyo, Japan), which contains 15% TFZ as the active ingredient and approximately 85% cyclohexanone, naphthalene, trimethylbenzene, naphtha, polyalkyl ether, other solvents, and surfactants. Although TFZ is considered safe for humans and animals, it causes mild hepatotoxicity in animals [3,4], especially in zebrafish and rodents. It induces peroxisomal proliferation and oxidative stress in the liver, leading to lipid peroxidation, mitochondrial dysfunction, and cell death. TFZ is a relatively non-toxic substance, and there are few reports of organ or health damage due to TFZ exposure in humans. We encountered a patient who developed severe impairment of consciousness and convulsions after ingesting Trifumin emulsion™ in an attempt to commit suicide. Here, we present the case of acute oral intoxication with TFZ and discuss the possible mechanisms and management.

## Case presentation

**Figure 1:**
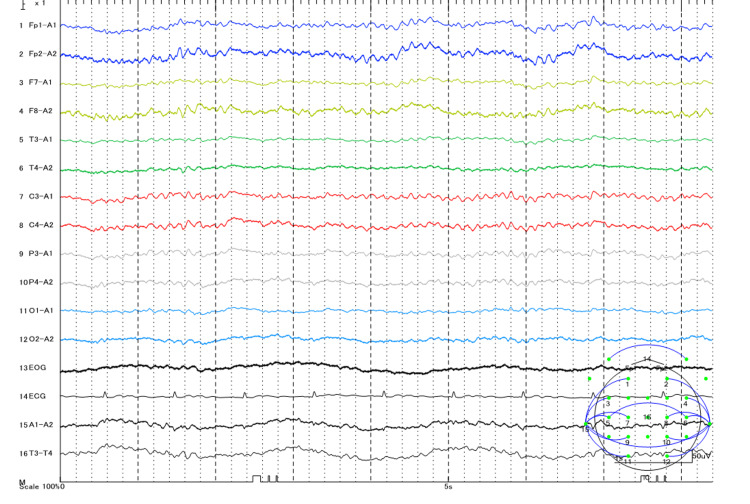
Continuous electroencephalogram monitoring revealed an alpha wave pattern.

A 70-year-old man (height: 165.0 cm, weight: 55.0 kg, with no significant medical history or allergy history, especially related to epilepsy) was promptly transported to our hospital with a disturbance of consciousness. He was barely conscious at the time of contact with emergency medical staff and reported having ingested Trifumin emulsion (approximately 50 mL) and alcohol (alcohol content 18%, 200 mL) a few hours before arrival at the hospital in an attempt to commit suicide. Upon admission, he presented with severe impairment of consciousness, categorized as Glasgow Coma Scale E1V1M1, and hypotension. His initial vital signs were as follows: blood pressure, 74/38 mmHg; heart rate, 64 beats/min; respiratory rate, 28 breaths/min; body temperature, 35.5°C; SpO2, 93% (room air); pupils, 5/5 -/-. The results of the arterial blood gas analysis at admission were as follows: pH 7.339; PaCO2 35 mmHg; HCO3- 18.3 mmol/L; and lactate 6.3 mmol/L. The baseline laboratory data at admission were as follows: Na 141 mmol/L; K 3.4 mmol/L; Cl 104 mmol/L; Anion Gap 18.7 mEq/L; Aspartate aminotransferase (AST) 39 U/L; ALT 31 U/L; Total Bilirubin 0.5 mg/dL; Blood Urea Nitrogen 26.3 mg/dL; Serum Creatinine 1.15 mg/dL. The estimated blood alcohol concentration calculated from the osmolar gap [5] was 42.1 mg/dL, which did not confirm acute alcohol intoxication [6]. Blood test results showed no obvious evidence of loss of consciousness or convulsions. Similarly, no obvious abnormal findings were observed on computed tomography. Although no convulsions were initially evident, the patient experienced generalized tonic-clonic convulsions a few hours after admission. Administration of lorazepam (2 mg) and midazolam (1 mg) initially controlled the convulsions; the patient required endotracheal intubation with propofol infusion (150 mg) for convulsion termination, and we administered levetiracetam (1000 mg/day) prophylactically as an anticonvulsant from day 1 of hospitalization. We performed continuous electroencephalogram (EEG) monitoring (Figure 1), which revealed an alpha wave. Neither obvious convulsions nor cramp discharge on EEG were observed after the first day of hospitalization. No recurrence of convulsions was observed after the completion of levetiracetam on the sixth day of hospitalization. On the fourth day of hospitalization, the patient was successfully weaned off mechanical ventilation and extubated. On the seventh day of hospitalization, he was transferred to the psychiatric department. The patient remained convulsion-free and demonstrated a favorable clinical course.

## Discussion

We present the first case of ingestion of Trifumin emulsion™ and alcohol, which resulted in severe impairment of consciousness and convulsions. Trifumin emulsion™ contains 15% TFZ as the active ingredient and approximately 85% other solvents and surfactants [2].

TFZ, an antifungal agricultural agent, is considered safe for humans and animals [3]. A report on the results of a yearly health examination of the personnel involved in TFZ production at a Takaoka plant (Japan) from May 1996 to May 2002 was prepared in accordance with the Japanese “Occupational Safety and Health Law” and has been submitted to the regulatory authority. Commercial production of TFZ began in 1985 at this plant. The health examination consisted of a physical examination, hematology, urine analysis, and blood chemistry. No adverse health effects attributable to chemical exposure were observed. In the TRIFLUMIZOLE 499-552 JMPR 2013 edition [7], it was reported that during the period covered, no events of acute poisoning by exposure or skin and/or eye irritation were observed.

Our patient presented with transient hypotension, severe impairment of consciousness, and delayed convulsions due to TFZ emulsion ingestion. The mechanism underlying TFZ-induced convulsions remains unclear; however, it is postulated to involve TFZ’s pharmacological properties.

An acute oral toxicity test of TFZ (purity: 98.7%) was conducted in rats. Mortality was observed in both male and female rats in the highest dose group. TFZ has been reported to possibly affect zebrafish development, accompanied by disturbances in oxidative stress, heat shock responses, inflammation, and lipid synthesis [4]. The toxicity signs of TFZ observed in the rats [7] included ataxia, hypotension, ventral position, lacrimation, urinary incontinence, decreased body temperature, heart rate, respiration rate, ptosis, and neurotoxicity. Hypotension and neurotoxicity can be explained by the effects of TFZ; however, impaired consciousness and convulsions observed in our patient are not included in these symptoms. Impaired consciousness may have resulted from ataxia, ptosis, and neurotoxicity. Hypotension and hypoxia may have caused convulsions. Measuring blood levels was also considered, but it was impossible to estimate how much ingestion would cause symptoms in the human body or whether it was safe. Moreover, the half-life of acute toxicity by oral ingestion in humans is unknown [7], and it could only be determined by attempting arousal daily.

Trifumin emulsion™ contains TFZ and substances such as emulsions [2], including cyclohexanone, naphthalene, and trimethylbenzene. These solvents might have contributed to the episode in the present case, including impairment of consciousness and convulsions. To date, most experimental animal studies on organic solvents [8] have aimed to determine their acute neurotoxic effects on the central nervous system (CNS). The acute toxic effects of solvent inhalation [9], as noted in animals, reflect those observed in humans and include narcosis, anesthesia, CNS depression, respiratory arrest, unconsciousness, and death. Organic solvents are substances known for their affinity for the nervous system. There is a potential causal relationship between epileptic convulsions and exposure to organic solvents [10]. Some individuals may be more sensitive to TFZ based on genetic factors or pre-existing nervous system conditions.

The interaction of TFZ with organic solvents and alcohol may also have caused severe consciousness impairment, disorientation, and convulsions in this patient. However, the osmolar gap that we tested was not high, and the alcohol consumed was not in a quantity considered harmful. Testimony and circumstantial evidence clearly indicate that he ingested TFZ emulsion and alcohol. We did not test for other toxic alcohols or poisons that can cause a mixed pattern of metabolic acidosis. However, there is no research on the interactions between TFZ, organic solvents, and alcohols. Based on this case report, it is necessary to further investigate pharmacological effects and interactions.

There are no specific antidotes or treatments for convulsions induced by either TFZ or solvents. Symptomatic therapy may be the only treatment option. In the present case, we managed to stop the seizures not by administering anticonvulsants but through sedation and waiting until the effects of the various substances wore off, leading to a good outcome.

## Conclusions

We present the first case report of a patient who developed severe impairment and convulsions after ingesting a TFZ emulsion and alcohol in a suicide attempt. There is a possibility of neurotoxicity due to TFZ alone; however, the potential for combined neurotoxicity due to organic solvents and alcohol cannot be ruled out. The neurotoxic effects noted in our patient were transient; symptomatic treatment involved waiting for the substances to be eliminated, resulting in good outcomes. This report highlights a case of TFZ intoxication and emphasizes the importance of further research to evaluate TFZ toxicity and its clinical implications.
